# Supplementary material for: The Latoia consocia Caterpillar Induces Pain by Targeting Nociceptive Ion Channel TRPV1
Source: Toxins (Basel). 2019 Nov 27;11(12):695. doi: 10.3390/toxins11120695 (PMC6950366; doi:10.3390/toxins11120695)
Supplement: Supplementary file 1 [file toxins-11-00695-s001.pdf]

Supplementary Materials: The *Latoia consocia* Caterpillar Induces Pain by Targeting Nociceptive Ion Channel TRPV1

Zhihao Yao, Peter Muiruri Kamau, Yalan Han, Jingmei Hu, Anna Luo, Lei Luo, Jie Zheng, Yuhua Tian and Ren Lai

Table 1. Defense mechanism-related genes found in *Latoia consocia* venom transcriptome.

Number	Gene ID	Description
1	TRINITY_DN83048_c1_g4_i3	Defense mechanisms
2	TRINITY_DN74008_c0_g2_i1	Defense mechanisms
3	TRINITY_DN80791_c0_g4_i2	Signal transduction mechanisms; Defense mechanisms
4	TRINITY_DN73676_c0_g1_i1	Extracellular structures; Defense mechanisms
5	TRINITY_DN77222_c0_g1_i1	Lipid transport and metabolism; Defense mechanisms
6	TRINITY_DN83852_c1_g1_i7	Signal transduction mechanisms; Defense mechanisms
7	TRINITY_DN79170_c2_g5_i1	Signal transduction mechanisms; Defense mechanisms
8	TRINITY_DN53275_c0_g1_i1	Signal transduction mechanisms; Defense mechanisms
9	TRINITY_DN80054_c2_g3_i1	Defense mechanisms
10	TRINITY_DN76642_c2_g2_i2	Signal transduction mechanisms; Defense mechanisms
11	TRINITY_DN75255_c0_g3_i1	Defense mechanisms
12	TRINITY_DN72565_c0_g1_i1	Signal transduction mechanisms; Defense mechanisms
13	TRINITY_DN82244_c0_g1_i1	Defense mechanisms
14	TRINITY_DN80536_c0_g1_i1	Defense mechanisms
15	TRINITY_DN66984_c0_g1_i1	Defense mechanisms
16	TRINITY_DN81172_c0_g1_i1	Signal transduction mechanisms; Defense mechanisms
17	TRINITY_DN79838_c3_g5_i1	Defense mechanisms
18	TRINITY_DN84195_c3_g1_i1	Defense mechanisms
19	TRINITY_DN78131_c0_g2_i4	Defense mechanisms

20	TRINITY_DN83853_c3_g2_i7	Defense mechanisms
21	TRINITY_DN83643_c1_g3_i1	Defense mechanisms
22	TRINITY_DN83475_c0_g2_i2	Signal transduction mechanisms; Defense mechanisms
23	TRINITY_DN83048_c1_g1_i6	Defense mechanisms
24	TRINITY_DN84195_c3_g4_i3	Defense mechanisms
25	TRINITY_DN84767_c1_g1_i3	Defense mechanisms
26	TRINITY_DN80385_c0_g1_i2	Defense mechanisms
27	TRINITY_DN80524_c0_g3_i1	Signal transduction mechanisms; Defense mechanisms
28	TRINITY_DN83475_c0_g1_i4	Signal transduction mechanisms; Defense mechanisms
29	TRINITY_DN81998_c0_g3_i1	Extracellular structures; Defense mechanisms
30	TRINITY_DN75666_c3_g1_i5	Defense mechanisms
31	TRINITY_DN82110_c0_g1_i6	Signal transduction mechanisms; Posttranslational modification, protein turnover, chaperones; Defense mechanisms
		Signal transduction mechanisms; Defense mechanisms
32	TRINITY_DN81269_c5_g2_i1	Defense mechanisms
33	TRINITY_DN77014_c1_g1_i2	Extracellular structures; Defense mechanisms
34	TRINITY_DN77967_c3_g1_i1	Defense mechanisms
35	TRINITY_DN34547_c0_g1_i1	Signal transduction mechanisms; Defense mechanisms
36	TRINITY_DN80253_c0_g2_i3	Defense mechanisms
37	TRINITY_DN67500_c0_g2_i2	Signal transduction mechanisms; Defense mechanisms
38	TRINITY_DN72730_c0_g1_i2	Defense mechanisms
39	TRINITY_DN78572_c1_g3_i8	Defense mechanisms
40	TRINITY_DN79935_c1_g1_i2	Signal transduction mechanisms; Defense mechanisms
41	TRINITY_DN76265_c1_g1_i1	Signal transduction mechanisms; Defense mechanisms
42	TRINITY_DN82697_c3_g6_i2	Signal transduction mechanisms; Defense mechanisms
43	TRINITY_DN79170_c2_g3_i1	Extracellular structures; Defense mechanisms

44	TRINITY_DN73206_c0_g1_i2	Defense mechanisms
45	TRINITY_DN82297_c5_g2_i1	Defense mechanisms
46	TRINITY_DN79000_c0_g1_i1	Defense mechanisms
47	TRINITY_DN76231_c0_g1_i5	Defense mechanisms
48	TRINITY_DN74127_c0_g1_i2	Defense mechanisms
49	TRINITY_DN82984_c4_g1_i2	Defense mechanisms
50	TRINITY_DN83795_c3_g1_i2	Defense mechanisms
51	TRINITY_DN75743_c0_g5_i1	Signal transduction mechanisms; Defense mechanisms
52	TRINITY_DN83261_c2_g2_i12	Signal transduction mechanisms; Defense mechanisms
53	TRINITY_DN81172_c1_g1_i2	Defense mechanisms
54	TRINITY_DN73759_c0_g1_i1	Signal transduction mechanisms; Defense mechanisms
55	TRINITY_DN75176_c8_g3_i1	Extracellular structures; Defense mechanisms
56	TRINITY_DN85012_c3_g3_i1	Signal transduction mechanisms; Defense mechanisms
57	TRINITY_DN77013_c0_g1_i2	Signal transduction mechanisms; Defense mechanisms
58	TRINITY_DN74738_c0_g1_i1	Signal transduction mechanisms; Defense mechanisms
59	TRINITY_DN75786_c3_g3_i1	Extracellular structures; Defense mechanisms
60	TRINITY_DN71515_c0_g1_i1	Defense mechanisms
61	TRINITY_DN71223_c0_g1_i1	Defense mechanisms
62	TRINITY_DN75742_c4_g1_i2	Signal transduction mechanisms; Defense mechanisms
63	TRINITY_DN80253_c0_g4_i1	Defense mechanisms
64	TRINITY_DN12228_c0_g1_i1	Defense mechanisms
65	TRINITY_DN67666_c0_g2_i1	Cell wall/membrane biogenesis; Defense mechanisms
66	TRINITY_DN82822_c0_g1_i3	Defense mechanisms
67	TRINITY_DN75759_c5_g1_i4	Signal transduction mechanisms; Defense mechanisms
68	TRINITY_DN82697_c3_g1_i5	Defense mechanisms
69	TRINITY_DN83643_c1_g2_i9	Extracellular structures; Defense mechanisms

70	TRINITY_DN73173_c0_g2_i1	Defense mechanisms
71	TRINITY_DN83643_c1_g1_i1	Extracellular structures; Defense mechanisms
72	TRINITY_DN85012_c5_g2_i2	Defense mechanisms
73	TRINITY_DN82618_c3_g2_i1	Defense mechanisms
74	TRINITY_DN72367_c0_g1_i1	Defense mechanisms
75	TRINITY_DN79060_c1_g1_i2	Signal transduction mechanisms; Defense mechanisms
76	TRINITY_DN80924_c3_g3_i1	Extracellular structures; Defense mechanisms
77	TRINITY_DN85012_c4_g1_i4	Signal transduction mechanisms; Defense mechanisms
78	TRINITY_DN65665_c0_g3_i1	Signal transduction mechanisms; Defense mechanisms
79	TRINITY_DN81608_c4_g1_i3	Defense mechanisms
80	TRINITY_DN80820_c2_g2_i2	Signal transduction mechanisms; Defense mechanisms
81	TRINITY_DN74013_c0_g1_i1	Defense mechanisms
82	TRINITY_DN82118_c3_g1_i2	Defense mechanisms
83	TRINITY_DN72121_c0_g4_i1	Defense mechanisms
84	TRINITY_DN79000_c0_g2_i2	Defense mechanisms
85	TRINITY_DN75743_c0_g2_i4	Extracellular structures; Defense mechanisms
86	TRINITY_DN80854_c2_g1_i4	Defense mechanisms
87	TRINITY_DN16636_c0_g1_i1	Signal transduction mechanisms; Defense mechanisms
88	TRINITY_DN83043_c0_g1_i2	Signal transduction mechanisms; Defense mechanisms
89	TRINITY_DN81172_c1_g2_i5	Defense mechanisms
90	TRINITY_DN70059_c0_g1_i1	Defense mechanisms
91	TRINITY_DN75738_c0_g1_i4	Extracellular structures; Defense mechanisms
92	TRINITY_DN85012_c3_g1_i1	Defense mechanisms
93	TRINITY_DN72609_c0_g1_i1	Signal transduction mechanisms; Defense mechanisms
94	TRINITY_DN78614_c2_g4_i2	Signal transduction mechanisms; Defense mechanisms
95	TRINITY_DN83852_c1_g3_i1	Defense mechanisms

96	TRINITY_DN73069_c0_g2_i1	Defense mechanisms
97	TRINITY_DN80864_c2_g1_i2	Signal transduction mechanisms; Defense mechanisms
98	TRINITY_DN75623_c3_g3_i1	Signal transduction mechanisms; Defense mechanisms
99	TRINITY_DN83852_c1_g2_i3	Extracellular structures; Defense mechanisms
100	TRINITY_DN73094_c0_g1_i2	Signal transduction mechanisms; Defense mechanisms
101	TRINITY_DN80924_c3_g5_i1	Defense mechanisms
102	TRINITY_DN83048_c1_g5_i1	Defense mechanisms
103	TRINITY_DN65896_c0_g1_i1	Signal transduction mechanisms; Defense mechanisms
104	TRINITY_DN73077_c0_g1_i2	Defense mechanisms
105	TRINITY_DN76198_c0_g1_i1	Signal transduction mechanisms; Defense mechanisms
106	TRINITY_DN80253_c0_g1_i3	Signal transduction mechanisms; Defense mechanisms
107	TRINITY_DN78366_c0_g1_i5	Signal transduction mechanisms; Defense mechanisms
108	TRINITY_DN83199_c0_g1_i4	Extracellular structures; Defense mechanisms
109	TRINITY_DN73173_c0_g1_i1	Defense mechanisms
110	TRINITY_DN77174_c0_g1_i1	Extracellular structures; Defense mechanisms
111	TRINITY_DN85012_c5_g1_i2	Defense mechanisms
112	TRINITY_DN75255_c0_g1_i1	Extracellular structures; Defense mechanisms
113	TRINITY_DN81998_c0_g2_i2	Defense mechanisms
114	TRINITY_DN82429_c0_g1_i4	Signal transduction mechanisms; Defense mechanisms
115	TRINITY_DN57746_c0_g1_i1	Signal transduction mechanisms; Defense mechanisms
116	TRINITY_DN80014_c0_g1_i4	Defense mechanisms
117	TRINITY_DN81258_c2_g1_i2	Defense mechanisms
118	TRINITY_DN72208_c0_g1_i1	Signal transduction mechanisms; Defense mechanisms
119	TRINITY_DN75819_c0_g1_i1	Defense mechanisms
120	TRINITY_DN67666_c0_g1_i1	Defense mechanisms
121	TRINITY_DN82590_c1_g2_i1	Defense mechanisms

122	TRINITY_DN80917_c2_g1_i2	Defense mechanisms
123	TRINITY_DN62754_c0_g1_i1	Defense mechanisms
124	TRINITY_DN81990_c1_g2_i3	Signal transduction mechanisms; Defense mechanisms
125	TRINITY_DN74529_c0_g1_i1	Extracellular structures; Defense mechanisms
126	TRINITY_DN72451_c0_g1_i1	Defense mechanisms
127	TRINITY_DN80758_c0_g1_i4	Signal transduction mechanisms; Defense mechanisms
128	TRINITY_DN68823_c0_g1_i1	Signal transduction mechanisms; Defense mechanisms
129	TRINITY_DN81930_c5_g3_i2	Signal transduction mechanisms; Defense mechanisms
130	TRINITY_DN82504_c0_g1_i3	Defense mechanisms
131	TRINITY_DN83170_c0_g1_i1	Defense mechanisms
132	TRINITY_DN80536_c0_g2_i2	Defense mechanisms
133	TRINITY_DN76908_c1_g1_i1	Defense mechanisms
134	TRINITY_DN82111_c0_g1_i6	Signal transduction mechanisms; Defense mechanisms
135	TRINITY_DN78614_c2_g2_i2	Defense mechanisms
136	TRINITY_DN81990_c1_g4_i1	Defense mechanisms
137	TRINITY_DN76459_c0_g1_i5	Defense mechanisms
138	TRINITY_DN81568_c0_g1_i2	Extracellular structures; Defense mechanisms
139	TRINITY_DN73339_c0_g1_i1	Extracellular structures; Defense mechanisms
140	TRINITY_DN70324_c0_g1_i1	Defense mechanisms
141	TRINITY_DN74744_c3_g1_i1	Signal transduction mechanisms; Defense mechanisms
142	TRINITY_DN76265_c0_g1_i3	Signal transduction mechanisms; Defense mechanisms
143	TRINITY_DN81608_c4_g2_i1	Signal transduction mechanisms; Defense mechanisms
144	TRINITY_DN76344_c3_g1_i1	Defense mechanisms
145	TRINITY_DN71223_c0_g2_i1	Defense mechanisms
146	TRINITY_DN84063_c1_g4_i2	Signal transduction mechanisms; Defense mechanisms
147	TRINITY_DN87531_c0_g1_i1	Signal transduction mechanisms; Defense mechanisms

148	TRINITY_DN82458_c0_g2_i1	Extracellular structures; Defense mechanisms
149	TRINITY_DN81998_c1_g1_i8	Defense mechanisms
150	TRINITY_DN75743_c0_g1_i3	Signal transduction mechanisms; Defense mechanisms
151	TRINITY_DN76809_c0_g3_i1	Extracellular structures; Defense mechanisms
152	TRINITY_DN71026_c0_g1_i2	Signal transduction mechanisms; Defense mechanisms
153	TRINITY_DN79582_c0_g1_i1	Extracellular structures; Defense mechanisms
154	TRINITY_DN85012_c5_g3_i2	General function prediction only; Defense mechanisms
155	TRINITY_DN78363_c0_g1_i5	Defense mechanisms
156	TRINITY_DN72121_c0_g1_i1	Defense mechanisms
157	TRINITY_DN75700_c0_g1_i6	Extracellular structures; Defense mechanisms
158	TRINITY_DN73339_c0_g2_i5	Signal transduction mechanisms; Defense mechanisms
159	TRINITY_DN76492_c1_g2_i1	Extracellular structures; Defense mechanisms
160	TRINITY_DN73738_c1_g1_i1	Signal transduction mechanisms; Defense mechanisms
161	TRINITY_DN76265_c2_g1_i4	Defense mechanisms
162	TRINITY_DN83526_c0_g1_i1	Defense mechanisms

Note: Sequencing data that support the findings of this study have been deposited in the NCBI Sequence Read Archive (SRA) and are accessible under the SRA accession PRJNA588657.
